# Acute fatty liver of pregnancy accompanied with disseminated intravascular coagulopathy and encephalopathy: A case report

**DOI:** 10.1002/ccr3.6485

**Published:** 2022-10-20

**Authors:** Saba Saleem Qazi, Shiza Danish, Anusha Akhai, Hania Khwaja, Muhammad Junaid Tahir, Mohammed Mahmmoud Fadelallah Eljack, Sabeeh Khawar Farooqui, Muhammad Sohaib Asghar

**Affiliations:** ^1^ Ziauddin Medical University Karachi Pakistan; ^2^ Lahore General Hospital Lahore Pakistan; ^3^ Teaching assistant, Faculty of Medicine and Health Sciences University of Bakht Alruda Ad Duwaym Sudan; ^4^ Dow University of Health Sciences–Ojha Campus Karachi Pakistan

**Keywords:** fatty liver, fetus, hepatology, jaundice, obstetrics, pathology

## Abstract

Acute fatty liver disease of pregnancy (AFLP) is a rare condition associated with other common liver manifestations such as hemolysis, elevated liver enzymes, and low platelets syndrome (HELLP). We present a 27‐year‐old pregnant woman who developed hepatic encephalopathy and DIC after being diagnosed with Acute fatty liver disease of pregnancy.

## Abstract 2

To reduce mortality, early AFLP diagnosis and management are critical. Non‐specific symptoms include anorexia, abdominal pain, and vomiting. The liver biopsy is highly sensitive. LFTs, PT, PTT, ammonia levels, and hypoglycemia are all common findings.

## 1 INTRODUCTION

Acute fatty liver disease of pregnancy (AFLP) is a life‐threatening condition in the same way as other causes of pathologies in the liver namely hemolysis, elevated liver enzymes, and low platelets (HELLP) syndrome.^1^ It is a life‐challenging disease that has shown a drastic rise in prevalence of about 1 to 3 cases per 10,000 deliveries occurring mostly in 3rd trimester marked by fatty infiltration of the liver leading to hepatic failure.^2^ The management of AFLP is prompt delivery of a fetus with improvements being noticed after 1–2 days of delivery.^3^ Early diagnosis is necessary to ensure better maternal and fetal outcomes.^4^ Clinical findings in AFLP can vary, and diagnosis is further complicated due to significant overlap with the clinical and biochemical markers of HELLP syndrome.^5^ The main finding related to AFLP includes infiltration of hepatocytes with fats including the pericentral zone while sparing the periportal hepatocyte.^6^ A higher rate of mortality is observed in developing countries due to a shortage of capacity for intensive care. Associated complications such as acute liver failure can arise if AFLP is left untreated. This is the stage where most of the AFLP is diagnosed.

To our knowledge, there is no case yet reported with the delivery of a live fetus in a patient with AFLP at 26 weeks of gestation. Hence, we are presenting a case of a pregnant 27‐year‐old female with AFLP. She was delivered a live male fetus due to prompt and early diagnosis despite being manifested with hepatic encephalopathy and disseminated intravascular coagulopathy (DIC).

## 2 CASE PRESENTATION

A 27‐year‐old woman (G3 P2+0), with three confirmed pregnancies and two previous deliveries, presented at 26 weeks of gestation in a tertiary care hospital with a history of fever, low platelets, and deranged liver function tests (LFTs) for 2 days. Her pregnancy was un‐booked (She was not getting her appointments and ultrasounds done as per the guideline recommendations for a pregnant woman), and the above‐mentioned findings were detected at a local clinic. Before this presentation, the patient was not on any medications.

On presentation to the hospital, physical examination revealed a well‐nourished and conscious woman who was oriented to time, place, and person. Her pulse rate was 82 beat per minute, blood pressure 120/80 mmHg, oxygen saturation of 95% at room air, blood glucose level 72 mg/dl, and ammonia 101 μmol/L. Abdominal examination revealed a height of the fundus corresponding to 26 cm. Neurological examination was non‐significant and the Glasgow Coma Scale (GCS) was 15/15. The rest of the physical examination was unremarkable.

She was started on empiric antibiotics and further investigations were done including Dengue NS1 antigen, LFTs, and Urine DR, to rule out other causes of her symptoms. A few hours into admission, the patient developed hypoglycemia for which she was intravenously given 25% dextrose.

**FIGURE 1:**
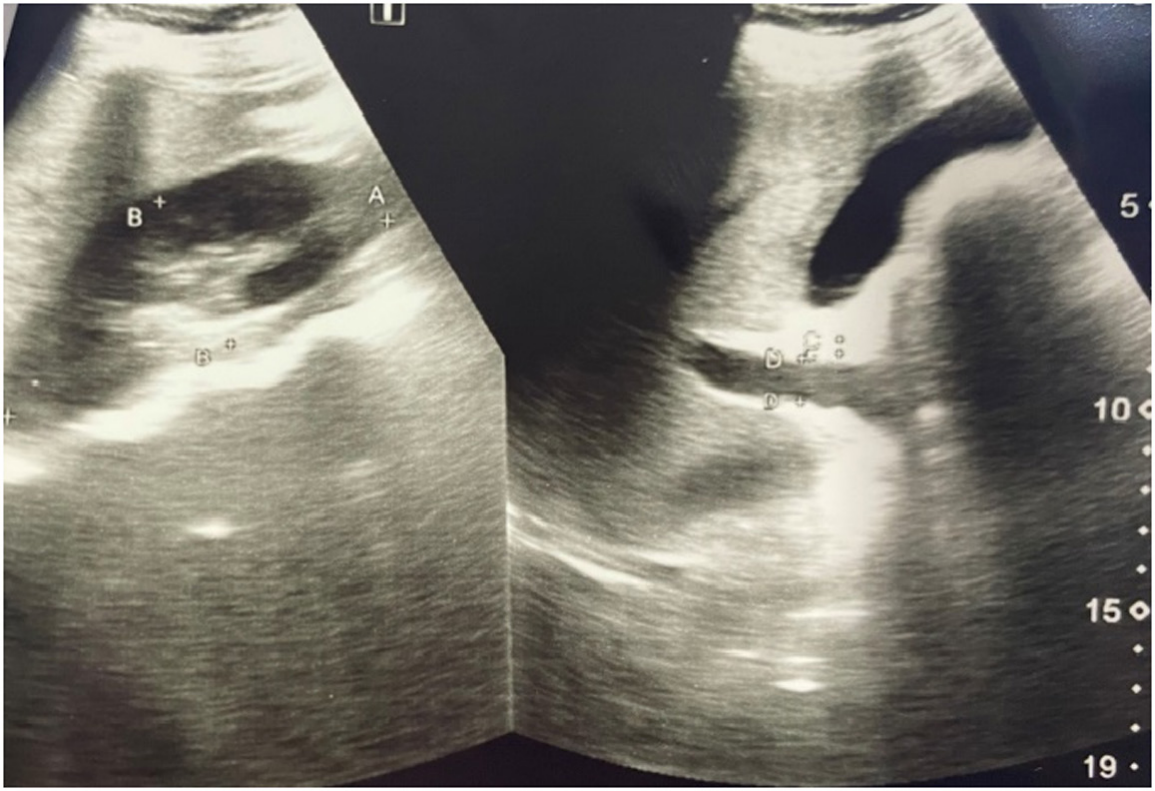
Ultrasound shows normal‐sized liver with fatty changes. No focal mass was noticed, and intrahepatic ducts were not dilated.

Due to the COVID‐19 endemic a chest X‐ray was performed. The patient was suspected of COVID‐19 but the results were in‐significant. Lung related symptoms improved as her AFLP was treated and atom nebulization was carried out for symptomatic relief. Atelectasis and other lung related symptoms resolved with treatment of AFLP. Ultrasound of the abdomen confirmed fatty changes with a normal‐sized hypoechoic liver sparing periportal zones ruling out HELLP (Figure 1). A provisional diagnosis of AFLP (and viral hepatitis) was made.

A normal sonogram of the gall bladder, pancreas, spleen, kidney, and urinary bladder was reported. The surface antigen test showed no reaction to HbsAg and HCV antigen, so ruling out viral hepatitis. A Swansea criterion was used to diagnose AFLP as presence of six or more terms mentioned in the criteria were noted in the patient which helped to make a confirmed diagnosis.

Twelve hours into admission, the patient complained of abdominal pain, vomiting, constipation, and headache, for which she was promptly transferred to the intensive care unit (ICU). The patient developed significant dyspnea with a total leukocyte count of 24.26 × 109/L, severe anemia, and a left‐sided consolidation on CXR.

She developed tachycardia with a pulse of 118/bpm. Fetal movements were positive. Her hemoglobin level dropped from 11.3 to 8.6 g/dl, with a prolonged APTT and INR, and her serum SGPT had risen to 3339 IU/L recorded in Table 1. The patient became markedly drowsy, irritable, and restless, and a diagnosis of Grade 1 (hepatic) encephalopathy was made, for which she was given lactulose. Later, patient progressed to Grade II hepatic encephalopathy, with a GCS of 13/15 and ammonia levels of 130 μmol/L.

**TABLE 1:** Average of 5 days of labs investigation recorded every 24 h for the patient

Investigations	Results (Average)	Unit	Normal range
PLT	66.5	×10^6^/mm^3^	157,000–371,000
APTT	40.7	S	30–40
PT	14.1	S	11 to 13.5
INR	1.27	‐	0.0–1.1
AST	1770	IU/L	5–43
ALP	262	IU/L	39.00–117.00
SGPT	594	IU/L	7–56
TB	2.72	mg/dl	0.1–1.2
DB	1.77	mg/dl	0 to 0.2

Abbreviations: ALP; Alkaline Phosphatase, APTT; Activated partial thromboplastin time, AST; Aspartate aminotransferase, DB; Direct Bilirubin, INR; International Normalized Ratio, PLT; Platelet count, PT; Prothrombin time, SGPT; serum glutamic‐pyruvic transaminase, TB; Total Bilirubin.

Vaginal delivery was chosen over a cesarean (C‐section) to prevent further deterioration of her condition as the patient was anemic and had DIC, which would have led to massive bleeding had we performed a C‐section. A live male fetus was delivered, with a poor APGAR score and was handed for resuscitation. Around 600–700 ml of clots were removed from the uterus.

An hour later, fresh bleeding was observed per vaginal examination. With a contracted uterus, her perineum was inspected for tears, balloon tamponade was performed. However, the patient continued to bleed and was shifted to the operation theater for uterine artery embolization. Her BP was 120/83 mmHg, pulse 120 bpm, and O2 saturation of 93% at room air. She was on continuous positive airway pressure (CPAP), and mannitol was administered to protect renal function after the vascular procedure.

The procedure successfully stopped her bleeding. The patient was drowsy, irritable, and not following commands, consistent with a progression to grade III hepatic encephalopathy, with a GCS of 11/15 and highest recorded ammonia levels of 156 μmol/L. Antibiotic treatment was continued.

The infant spent 2 days in the intensive care unit (ICU) before dying due to pre‐maturity and improper lung development.

**FIGURE 2:**
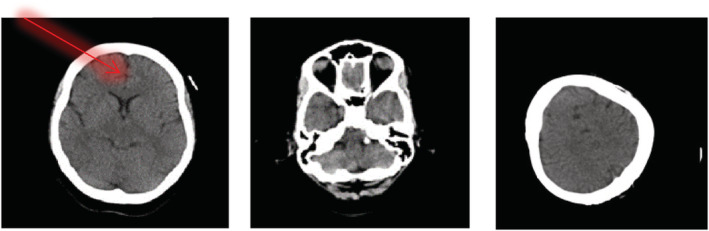
CT head without contrast. Normal Ventricular system. Arrow showing no midline shift noted. No hemorrhage, tumor, or infarction noted.

Over the course of the next 2 days, the patient's condition improved steadily. CT of the brain showed no cerebral edema (Figure 2).

Approximately 2 months later, the patient returned to our facility with a complaint of lower abdominal pain. On examination, her uterus was bulky, with a thickened endometrium and necrotic debris sloughing off through the vagina. She had developed uterine necrosis; a late and rare complication of the uterine artery embolization that had been performed for her postpartum hemorrhage. As a result, a total abdominal hysterectomy was performed, after which she was discharged from the hospital.

## 3 DISCUSSION

Liver injury during pregnancy has many causes including but not limited to intrahepatic cholestasis, cholelithiasis, viral hepatitis, pre‐eclampsia with or without HELLP syndrome, and AFLP. Intrahepatic cholestasis of pregnancy (ICP) may present during the third trimester but it is mainly characterized by pruritis in the absence of a skin rash with abnormal LFTs (elevated transaminase and bile acids) and usually resolves after birth; however, serum bilirubin concentration is rarely higher than 6 mg/dl. Cholelithiasis may occur at any time during pregnancy and is accompanied by pain in the right upper quadrant, and fever, and ultrasonography (USG) is usually diagnostic.^7^ Acute viral hepatitis in pregnancy presents as a systemic illness with fever, nausea, vomiting, fatigue, jaundice, dark urine, and pale stools, and markedly elevated aminotransferase concentrations (>500 U/L).

The incidence of HELLP syndrome is three times as much as that of AFLP.^8^ The symptoms of our patient were initially indicative of both HELLP and AFLP; nausea, vomiting, dyspnea, epigastric abdominal pain, anorexia, jaundice, and hence supportive treatment for AFLP and HELLP Syndrome was started. Further laboratory investigations indicated severe coagulopathy, raised APTT, elevated serum transaminase and bilirubin levels, hypoglycemia, an elevated ammonia value, and a low albumin level, favoring the diagnosis of AFLP over HELLP syndrome.

In AFLP, there is an inherited enzyme deficiency in beta‐oxidation, due to a defect in the enzyme LCHAD (long‐chain hydroxy acyl‐coenzyme A dehydrogenase) predisposing them to AFLP resulting in progressive lipid accumulation within the hepatocytes. However, there are other hypotheses that have been reported linking risk factors such as multiple gestations and use of non‐steroidal anti‐inflammatory drugs (NSAIDS) with AFLP. A hypothesis is made that multiple gestations enhances the risk for AFLP due to the over‐production of fatty acid metabolites.^9^ Furthermore, use of NSAIDs causes the inhibition of tri‐functional protein and eventually leading to long‐chain fatty acid oxidation in mitochondria, which may lead to development of AFLP.^10^ All these risk factors are associated with development of AFLP without the presence of a mutation in LCHAD gene.

Lastly, extrahepatic complications delayed maternal recovery for up to 4 weeks after delivery. These trends in earlier studies make this case report noteworthy as our patient presented to us with hepatic encephalopathy, acute renal failure, and DIC but against all the odds improved a week before delivery.

## 4 CONCLUSION

Early diagnosis and prompt management are essential to prevent maternal and perinatal morbidity and mortality. AFLP can be managed with early and prompt diagnosis through laboratory tests to reduce complications. Further studies can be conducted to devise a relationship between factors that can lead to termination of the pregnancy in patients with hepatic encephalopathy and DIC so neonatal deaths can be reduced, and a solution can be stamped with regards to AFLP.

## AUTHOR CONTRIBUTIONS

Saba Saleem Qazi and Hania Khwaja involved in conceptualization. Anusha Akhai and Shiza Danish involved in data curation. Hania Khwaja involved in formal analysis. Shiza Danish involved in investigation. Saba saleem Qazi, Sabeeh Khawar Farooqui, Hania Khwaja, and Mohammed Mahmmoud Fadelallah Eljack involved in methodology. Sabeeh Khawar Farooqui, Saba Saleem Qazi, Anusha Akhai, Muhammad Sohaib Asghar, Muhammad Junaid Tahir, and Mohammed Mahmmoud Fadelallah Eljack involved in writing (main draft). Muhammad Sohaib Asghar, and Muhammad Junaid Tahir involved in writing (review and editing).

## FUNDING INFORMATION

This study received no external funding.

## CONSENT

Written informed consent was obtained from the patient to publish this report in accordance with the journal's patient consent policy.

## CONSENT FOR PUBLICATION

An institutional approval letter was granted by Ziauddin Medical University, Karachi, Pakistan, for this case report.

## Data Availability

The data that support the findings of this study are available with the corresponding author upon reasonable request.

## References

[1] Vigil‐De GP . Acute fatty liver and HELLP syndrome: two distinct pregnancy disorders. Int J Gynaecol Obstet. 2001;73(3):215‐220. 11376667 10.1016/s0020-7292(01)00364-2

[2] Naoum EE , Leffert LR , Chitilian HV , Gray KJ , Bateman BT . Acute fatty liver of pregnancy: pathophysiology, anesthetic implications, and obstetrical management. Anesthesiology. 2019;130(3):446‐461. 30707120 10.1097/ALN.0000000000002597 PMC7037575

[3] Ziki E , Bopoto S , Madziyire MG , Madziwa D . Acute fatty liver of pregnancy: a case report. BMC Pregnancy Childbirth. 2019;19(1):259. 31331287 10.1186/s12884-019-2405-5 PMC6647299

[4] Vora KS , Shah VR , Parikh GP . Acute fatty liver of pregnancy: a case report of an uncommon disease. Indian J Crit Care Med. 2009;13:34‐36. 19881179 10.4103/0972-5229.53115 PMC2772253

[5] English N , Rao J . Acute fatty liver of pregnancy with hypoglycaemia, diabetes insipidus and pancreatitis, preceded by intrahepatic cholestasis of pregnancy. BMJ Case Rep. 2015;2015:bcr2015209649. 10.1136/bcr-2015-209649 PMC4401975 25878236

[6] Liu J , Ghaziani TT , Wolf JL . Acute fatty liver disease of pregnancy: updates in pathogenesis, diagnosis, and management. Am J Gastroenterol. 2017;112(6):838‐846. 28291236 10.1038/ajg.2017.54

[7] Gangwar R , Dayal M , Dwivedi M , Ghosh U . No title. J Obstet Gynecol India. 2011;61(1):57‐61.

[8] Pereira SP , O'Donohue J , Wendon J , Williams R . Maternal and perinatal outcome in severe pregnancy‐related liver disease. Hepatology. 1997;26(5):1258‐1262. 9362370 10.1002/hep.510260525

[9] Davidson KM , Simpson LL , Knox TA , D'Alton ME . Acute fatty liver of pregnancy in triplet gestation. Obstet Gynecol. 1998;91(5 Pt 2):806‐808. 9572168 10.1016/s0029-7844(97)00477-8

[10] Saygan‐Karamürsel B , Kizilkiliç‐Parlakgümüş A , Deren O , Onderoğlu L , Durukan T . Acute fatty liver of pregnancy after aspirin intake. J Matern Neonatal Med. 2004;16(1):65‐66. 10.1080/14767050412331283120 15370086

